# Oridonin Attenuates Cisplatin-Induced Acute Kidney Injury via Inhibiting Oxidative Stress, Apoptosis, and Inflammation in Mice

**DOI:** 10.1155/2022/3002962

**Published:** 2022-04-16

**Authors:** Hyemin Gu, Mi-Gyeong Gwon, Jong Hyun Kim, Jaechan Leem, Sun-Jae Lee

**Affiliations:** ^1^Department of Pathology, School of Medicine, Daegu Catholic University, Daegu 42472, Republic of Korea; ^2^Department of Biochemistry, School of Medicine, Daegu Catholic University, Daegu 42472, Republic of Korea; ^3^Department of Immunology, School of Medicine, Daegu Catholic University, Daegu 42472, Republic of Korea

## Abstract

The use of cisplatin, a chemotherapy drug, is often limited due to its renal side effects such as acute kidney injury (AKI). However, there are no validated medications to prevent or treat cisplatin-induced AKI. Oridonin is the major bioactive component of *Isodon rubescens* (*Rabdosia rubescens*) and exhibits anticancer, antioxidative, and anti-inflammatory effects. Recent studies have shown that oridonin alleviated a variety of inflammatory diseases, including renal diseases, in rodents. This study was aimed at investigating the potential renoprotective effect of oridonin on cisplatin-induced AKI. Male C57BL/6 mice were administered with cisplatin (20 mg/kg) with or without oridonin (15 mg/kg). Oridonin administration to mice after cisplatin injection attenuated renal dysfunction and histopathological changes. Upregulation of tubular injury markers was also suppressed by oridonin. Mechanistically, oridonin suppressed lipid peroxidation and reversed the decreased ratio of reduced to oxidized glutathione in cisplatin-injected mice. The increase in cisplatin-induced apoptosis was also alleviated by the compound. Moreover, oridonin inhibited cytokine overproduction and attenuated immune cell infiltration in cisplatin-injected mice. Altogether, these data demonstrated that oridonin alleviates cisplatin-induced kidney injury via inhibiting oxidative stress, apoptosis, and inflammation.

## 1. Introduction

Acute kidney injury (AKI) is characterized by a sudden decrease in renal function and is one of the major global health problems [1]. The severity of AKI is positively associated with in-hospital mortality, length of hospital stay, and medical care costs [2]. In the long term, AKI is also related to an increased risk of cardiovascular events, progression to chronic kidney disease, and long-term mortality [2]. The primary causes of AKI include renal ischemia-reperfusion, sepsis, and nephrotoxins. Among them, nephrotoxic drugs are increasingly considered as substantial contributors to AKI in hospitalized patients [3]. Cisplatin is a widely used chemotherapy drug to treat many types of cancer, including breast, testicular, and ovarian cancers [4]. Although the drug has potent antitumor effects, its serious side effects often limit its clinical use [4]. Nephrotoxicity is an important side effect of cisplatin therapy, and the nephrotoxic effects of cisplatin are dose-dependent and cumulative [5]. Unfortunately, despite the limited clinical application of cisplatin due to renal side effects, there are no validated drugs that prevent or treat its nephrotoxicity.

Oridonin is a diterpenoid compound found in *Isodon rubescens* (*Rabdosia rubescens*) [6]. Accumulating evidence suggest that oridonin has potent anticancer, antioxidative, and anti-inflammatory activities [7–9]. Although many studies have focused on elucidating the antitumor effect of oridonin [7], emerging evidence suggest that the compound inhibits renal ischemia-reperfusion injury in mice via suppressing inflammatory pathways [10, 11]. Moreover, oridonin attenuated diabetes-associated renal inflammation and injury in rats [12], suggesting that the compound has a protective action against both acute and chronic kidney injury. However, it has not yet been determined whether oridonin has a beneficial action on cisplatin nephrotoxicity. Thus, in the current study, we examined the effect of oridonin on cisplatin-induced kidney injury and explored the mechanism.

## 2. Materials and Methods

### 2.1. Animal Experiments

Male C57BL/6 mice were obtained from HyoSung Science (Daegu, Korea) and maintained at a temperature of at 20-24°C and humidity of 60-70%. The mice were grouped into three groups (*n* = 8 per group): the control group, the CP group, and the CP+Ori group. The CP group received a single intraperitoneal injection of cisplatin (20 mg/kg; Sigma-Aldrich, St. Louis, MO, USA). The CP+Ori group was given an intraperitoneal administration of oridonin (15 mg/kg; dissolved in DMSO; Sigma-Aldrich) daily for 3 consecutive days, starting from 1 hour after cisplatin injection. The control group received intraperitoneal injections of an equal volume of DMSO daily for 3 consecutive days. All mice were sacrificed 72 hours after a single dose of cisplatin. The doses of oridonin and cisplatin were selected based on the results of previous studies [10, 13]. All animal procedures were approved by the Institutional Animal Care and Use Committee of the Daegu Catholic University Medical Center (DCIAFCR-200626-12-Y).

### 2.2. Plasma and Tissue Biochemical Assays

Serum creatinine and blood urea nitrogen (BUN) levels were assessed using an automatic analyzer (Hitachi, Osaka, Japan). Serum tumor necrosis factor-*α* (TNF-*α*) and interleukin-6 (IL-6) levels were analyzed using ELISA kits (R&D Systems, Minneapolis, MN, USA). Malondialdehyde (MDA) levels were analyzed using a MDA assay kit (Sigma-Aldrich). Reduced glutathione (GSH) and oxidized glutathione (GSSG) levels were measured using a GSH assay kit (Enzo Life Sciences, Farmingdale, NY, USA). All analyses were conducted following the manufacturers' protocols.

### 2.3. Histological and Immunohistochemistry (IHC) Staining

Formalin-fixed tissues were dehydrated, cleared, and embedded in paraffin. The tissue blocks were sectioned and stained with hematoxylin and eosin (H&E) or periodic acid-Schiff (PAS). Tubular injury score was assessed in 5 randomly selected fields per sample, as previously described [14, 15]. For IHC, primary antibodies against neutrophil gelatinase-associated lipocalin (NGAL; Santa Cruz Biotechnology, Santa Cruz, CA, USA), kidney injury molecule-1 (KIM-1; Santa Cruz Biotechnology), 4-hydroxy-2-nonenal (4-HNE; Invitrogen, Carlsbad, CA, USA), F4/80 (Santa Cruz Biotechnology), and CD4 (Novus Biologicals, Littleton, CO, USA) antibodies were used. Mouse IgG1 isotype control antibody (R&D Systems) was used as a primary antibody for negative control. Positive areas were examined in 5 randomly selected fields at 400x magnification per sample using a computerized image analyzer (i-Solution DT software; IMT i-Solution, Coquitlam, BC, Canada), and the results were presented as percentage of the positively stained area with respect to the total area analyzed. Positive cells were examined in 10 randomly selected fields at 1000x magnification per sample.

### 2.4. Western Blot Analysis

Western blot analysis was conducted using primary antibodies against cleaved caspase-3 (Cell Signaling Technology, Danvers, MA, USA), caspase-3 (Cell Signaling Technology), TNF-*α* (Abcam, Cambridge, MA, USA), and glyceraldehyde-3-phosphate dehydrogenase (GAPDH; Cell Signaling Technology), as previously described [15]. Protein bands were visualized using enhanced chemiluminescence reagents (Thermo Fisher Scientific, Waltham, MA, USA).

### 2.5. qPCR Analysis

Total RNA isolation was performed using the TRIzol reagent (Sigma-Aldrich). Total RNA was reverse-transcribed into cDNA using the PrimeScript RT Reagent Kit (TaKaRa, Tokyo, Japan). For qPCR analysis, the Power SYBR Green PCR Master Mix (Thermo Fisher Scientific) and the Thermal Cycler Dice Real Time System III (TaKaRa) were used. Primers are shown in Table 1. GAPDH was used as a reference gene. Data were analyzed using 2^-*ΔΔ*CT^ method.

### 2.6. TUNEL Assay

Apoptosis was examined using a TUNEL assay kit (Roche Diagnostics, Indianapolis, IN, USA) following the manufacturer's protocol. Briefly, the kidney sections were deparaffinized, rehydrated, and permeabilized for 30 min at room temperature with proteinase K in 10 mM Tris-HCl, pH 7.4. After washing, the sections were incubated in the TUNEL reaction mixture for 1 h at 37°C. DAPI was used for nuclear staining. Positive cells were examined in 10 randomly selected fields at 600x magnification per sample.

### 2.7. Statistical Analysis

Data are presented as mean ± SEM. Differences among the groups were analyzed with one-way ANOVA and Bonferroni's post hoc tests. A *p* value less than 0.05 was considered statistically significant.

## 3. Results

### 3.1. Oridonin Ameliorated Renal Dysfunction and Structural Damage in Cisplatin-Injected Mice

**Figure 1:**
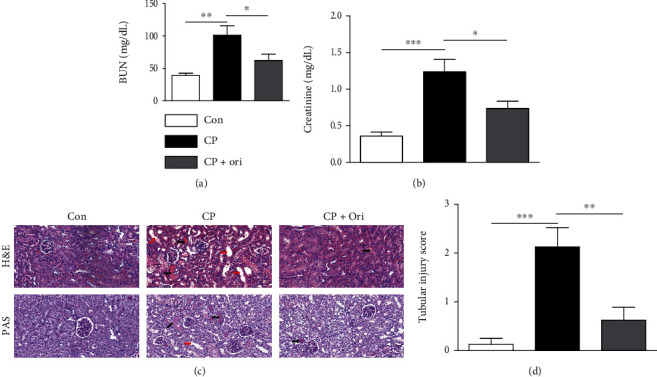
Effect of oridonin on renal function and histological abnormalities in cisplatin-injected mice. (a) Serum creatinine levels. (b) BUN levels. (c) H&E and PAS staining of kidney sections. Scale bar = 40 *μ*m. Red arrows indicate tubular dilatation. Black arrows indicate cast deposition in the lumens of tubules. (d) Tubular injury score. *n* = 8 per group. ^∗^*p* < 0.05, ^∗∗^*p* < 0.01, and ^∗∗∗^*p* < 0.001.

To assess renal function, serum creatinine and BUN levels, indicators of renal function [16], were measured in all experimental groups. Intraperitoneal injection of cisplatin increased serum levels of the indicators (Figures 1(a) and 1(b)). Cisplatin-injected mice exhibited significant tubular damage, including tubular dilatation and cast formation, as shown by histological examination (Figures 1(c) and 1(d)). However, these changes were significantly attenuated by oridonin (Figures 1(a)–1(d)).

**Figure 2:**
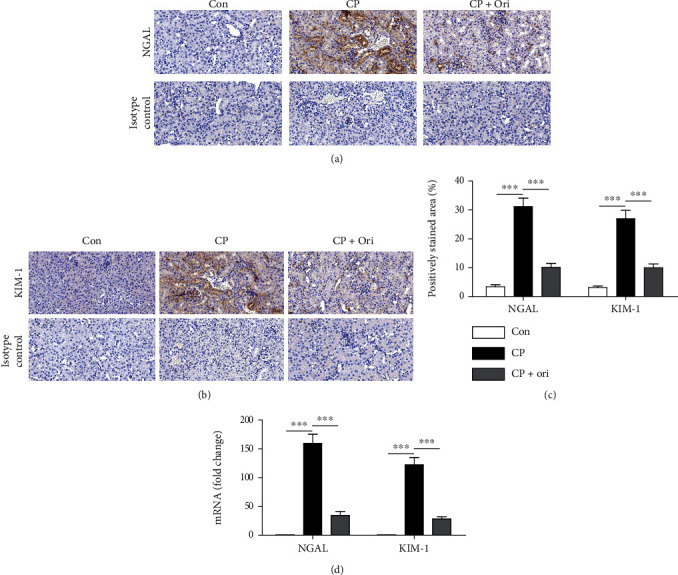
Effect of oridonin on NGAL and KIM-1 expression. (a) IHC staining for NGAL. Scale bar = 40 *μ*m. (b) IHC staining for KIM-1. Scale bar = 40 *μ*m. (c) Quantification of positive staining for NGAL and KIM-1. (d) Relative mRNA levels of NGAL and KIM-1. *n* = 8 per group. ^∗∗∗^*p* < 0.001.

Renal tubular injury is a hallmark of cisplatin-induced kidney injury [17]. To more clearly assess the action of oridonin on cisplatin-induced tubular injury, the renal expression of NGAL and KIM-1, tubular injury markers [18], was examined using IHC staining. Expression of the markers was elevated after cisplatin injection (Figures 2(a)–2(c)). Moreover, their mRNA levels were also increased (Figure 2(d)). However, the upregulation of the markers was significantly inhibited by oridonin (Figures 2(a)–2(d)).

### 3.2. Oridonin Suppressed Oxidative Stress

**Figure 3:**
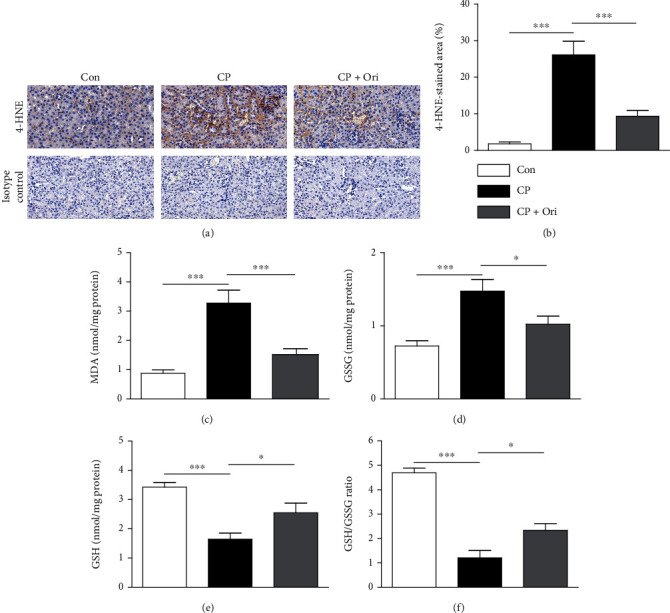
Effect of oridonin on oxidative stress. (a) IHC staining for 4-HNE. Scale bar = 40 *μ*m. (b) Quantification of positive staining for 4-HNE. (c) Renal MDA levels. (d) Renal GSSG levels. (e) Renal GSH levels. (f) GSH/GSSG ratios. *n* = 8 per group. ^∗^*p* < 0.05 and ^∗∗∗^*p* < 0.001.

Oxidative stress is a crucial mechanism of cisplatin nephrotoxicity [19]. Therefore, we examined renal expression of 4-HNE, a lipid peroxidation product [20], in all experimental groups. Cisplatin injection increased 4-HNE expression in the renal cortex compared to control group (Figures 3(a) and 3(b)). Amounts of MDA, another lipid peroxidation product [20], were also increased after cisplatin injection (Figure 3(c)). However, oridonin significantly lowered the increased levels of lipid peroxidation products induced by cisplatin (Figures 3(a)–3(c)). In addition, after cisplatin injection, GSSG levels (Figure 3(d)) were increased in kidneys, while GSH levels (Figure 3(e)) and GSH/GSSG ratios (Figure 3(f)) were decreased. These alterations were significantly reversed by oridonin (Figures 3(d)–3(f)), indicating that the compound suppressed cisplatin-induced oxidative stress.

### 3.3. Oridonin Attenuated Apoptotic Cell Death

**Figure 4:**
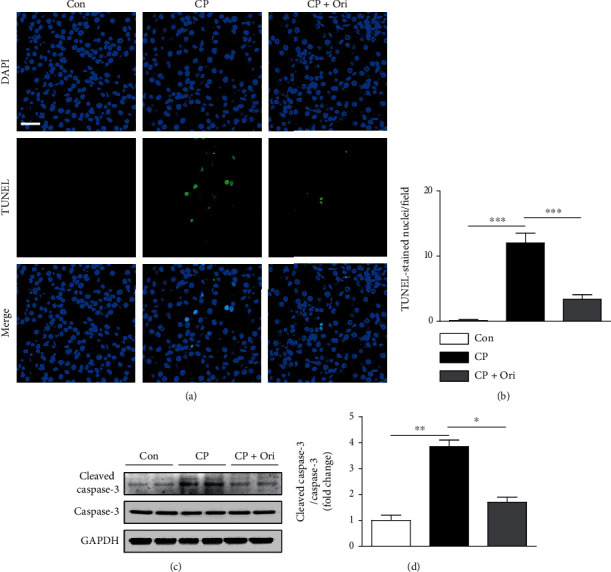
Effect of oridonin on tubular cell apoptosis. (a) TUNEL assay on kidney sections. Scale bar = 10 *μ*m. To detect nuclei, DAPI was used. (b) Number of TUNEL-stained nuclei per field. (c) Western blotting of cleaved caspase-3. (d) Quantification of Western blot results for cleaved caspase-3. *n* = 8 per group. ^∗^*p* < 0.05, ^∗∗^*p* < 0.01, and ^∗∗∗^*p* < 0.001.

Because apoptosis of tubular cells is frequently observed in cisplatin-induced kidney injury [5], TUNEL assay was conducted to assess the effect of oridonin on apoptosis. Cisplatin injection increased the number of TUNEL-positive cells in the kidney (Figures 4(a) and 4(b)). Caspase-3 cleavage was also increased (Figures 4(c) and 4(d)). However, cisplatin-induced apoptosis was significantly inhibited by oridonin (Figures 4(a)–4(d)).

### 3.4. Oridonin Inhibited Inflammatory Responses

**Figure 5:**
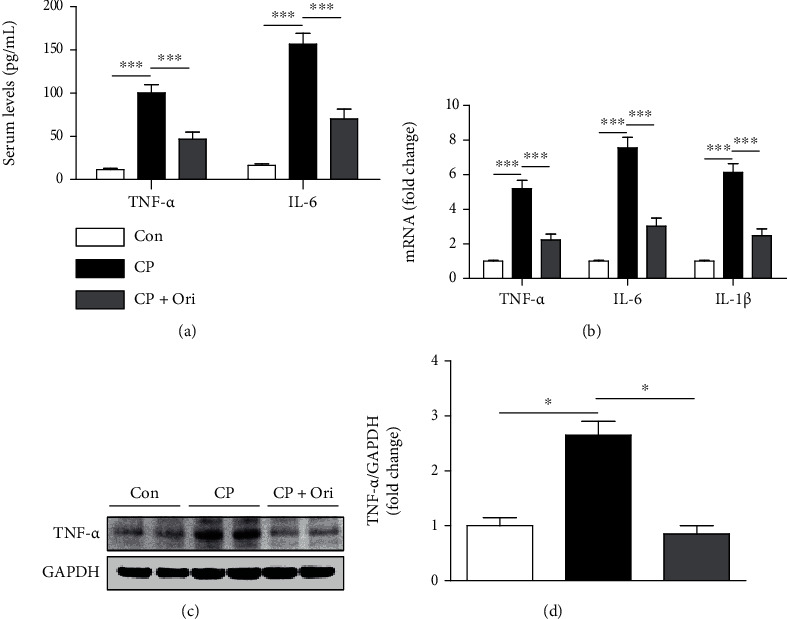
Effect of oridonin on cytokine production. (a) Serum levels TNF-*α* and IL-6. (b) Relative mRNA levels of TNF-*α*, IL-6, and IL-1*β*. (c) Western blotting of TNF-*α*. (d) Quantification of Western blot results for TNF-*α*. *n* = 8 per group. ^∗^*p* < 0.05 and ^∗∗∗^*p* < 0.001.

Inflammation also contributes to the pathophysiology of cisplatin nephrotoxicity [5]. Cisplatin-injected mice had elevated serum TNF-*α* and IL-6 levels compared to controls (Figure 5(a)). Renal levels of TNF-*α*, IL-6, and IL-1*β* mRNA was also increased after cisplatin injection (Figure 5(b)). Increase protein levels of TNF-*α* were also detected by Western blot analysis (Figures 5(c) and 5(d)). However, oridonin significantly lowered serum and tissue levels of the cytokines (Figures 5(a)–5(d)).

**Figure 6:**
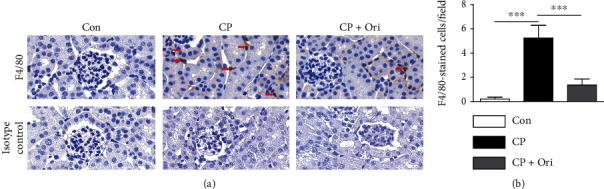
Effect of oridonin on macrophage infiltration. (a) IHC staining for F4/80. Scale bar = 20 *μ*m. Red arrows indicate positive cells. (b) Number of F4/80-positive cells per field. *n* = 8 per group. ^∗∗∗^*p* < 0.001.

**Figure 7:**
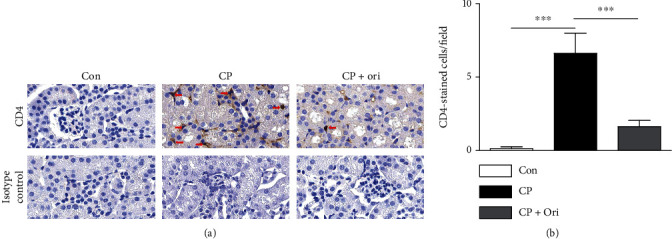
Effect of oridonin on CD4^+^ T cell infiltration. (a) IHC staining for CD4. Scale bar = 20 *μ*m. Red arrows indicate positively stained cells. (b) Number of CD4-positive cells per field. *n* = 8 per group. ^∗∗∗^*p* < 0.001.

Because immune cells infiltrate into the kidney and secrete large amounts of cytokines during cisplatin-induced kidney injury [5], we next performed IHC staining with antibodies against F4/80 and CD4 to detect macrophages and CD4^+^ T cells, respectively. The number of macrophages was increased after cisplatin injection but was significantly alleviated by oridonin (Figures 6(a) and 6(b)). Administration of oridonin also decreased the number of CD4^+^ T cells in cisplatin-injected mice (Figures 7(a) and 7(b)).

## 4. Discussion

In the current study, we demonstrated the therapeutic effect of oridonin on cisplatin-induced kidney injury. Mechanistically, oridonin inhibited oxidative stress, tubular cell apoptosis, and inflammation in cisplatin-injected mice.

Early studies on the function of oridonin have mainly focused on its anticancer effect [7]. Indeed, oridonin has been shown to exert anticancer activity on many types of cancers [21–23]. However, subsequent studies suggest that in addition to its anticancer effect, oridonin has several other favorable effects including antioxidative and anti-inflammatory effects [8, 9]. Therefore, we hypothesized that oridonin may have a beneficial effect on cisplatin nephrotoxicity. In the current study, administration of oridonin ameliorated renal dysfunction and histopathological alterations, suggesting that the compound has a therapeutic action on cisplatin-induced kidney injury. Besides nephrotoxic medications, ischemia-reperfusion injury is also a major cause of AKI [24]. Recent studies have demonstrated the protective effect of oridonin on renal ischemia-reperfusion injury [10, 11]. These findings suggest that the beneficial action of oridonin is not limited to cisplatin-induced AKI but may also be applied to other types of AKI.

Oxidative stress has been known to play a critical role in various diseases, including cardiovascular diseases, metabolic diseases, and neurodegenerative diseases [25]. Animal studies have shown that cisplatin nephrotoxicity is alleviated by administration of various antioxidants, such as coenzyme Q10 [26], vitamin C [27], vitamin E [28], resveratrol [29], and melatonin [30], suggesting that oxidative stress is also an important mechanism of cisplatin nephrotoxicity. Importantly, various natural compounds have antioxidative activity [31, 32]. Antioxidative effect of oridonin has also been reported in several studies [8, 9]. Oridonin suppressed reactive oxygen species generation in lipopolysaccharide- (LPS-) treated human renal tubular epithelial cells [33]. In the present study, administration of oridonin decreased the amounts of 4-HNE and MDA in kidneys of cisplatin-injected mice. These molecules are well-known lipid peroxidation products and have been shown to be increased in cisplatin nephrotoxicity [34, 35]. Moreover, cisplatin injection lowered the GSH/GSSG ratio, indicating increased oxidative stress [36, 37]. However, oridonin significantly reversed the decreased GSH/GSSG ratio. Therefore, the therapeutic action of oridonin on cisplatin-induced kidney injury is possibly attributed to its antioxidative effect.

Tubular cell apoptosis is frequently observed and is mainly caused by oxidative stress in cisplatin-induced kidney injury [38]. In the current study, cisplatin injection resulted in caspase-3 activation and apoptosis, which were inhibited by oridonin. Cisplatin can activate proapoptotic proteins, which cause the translocation of cytochrome c into the cytoplasm [19]. Then, the mediator induces the assembly of a multiprotein complex, resulting in activation of executioner caspases. Therefore, our data suggest that oridonin attenuated cisplatin-induced apoptosis through suppressing caspase-3 pathway. Consistent with our findings, oridonin inhibited hypoxia-induced apoptosis in a rat cardiomyoblast cell line [39]. Oridonin also protected human keratinocytes and dermal fibroblasts against hydrogen peroxide-induced apoptosis [40, 41]. Furthermore, the compound inhibited hepatocyte apoptosis to ameliorate acute liver injury in mice [42].

Excessive cytokine secretion and immune cell infiltration are characteristic features of cisplatin-induced AKI [43–45]. Oridonin inhibited cisplatin-induced systemic and renal inflammation, as evidenced by reductions in both serum and renal levels of cytokines. Increased infiltration of macrophages and CD4^+^ T cells was also alleviated by oridonin. Consistently, emerging evidence suggest that the beneficial action of oridonin on renal ischemia-reperfusion injury is associated with suppression of macrophage-mediated inflammation [10, 11]. It has been also reported that oridonin can modulate the activation and proliferation of T cells to alleviate inflammatory diseases such as inflammatory bowel disease [46, 47] and asthma [48]. In addition, oridonin inhibited LPS-induced cytokine production in human gingival fibroblasts [49] and mouse endometrial epithelial cells [50]. Inflammatory responses in IL-1*β*-stimulated human osteoarthritis chondrocytes were also suppressed by oridonin [51].

Oridonin has broad potential for drug development due to its wide range of pharmacological activities [8, 52]. However, oridonin has low solubility and poor bioavailability, which limits its clinical application. Therefore, much effort should be focused on the development of strategies, such as structural modification and new dosage form, to overcome these shortcomings [52].

In conclusion, we showed that oridonin ameliorated cisplatin-induced kidney injury in mice and that its therapeutic effect was due to attenuation of oxidative stress, apoptosis, and inflammation. The compound has been shown to increase the susceptibility of cancer cells to chemotherapy drugs including cisplatin [53, 55]. Therefore, oridonin may be a useful therapeutic agent for AKI in cancer patients undergoing chemotherapy.

## Figures and Tables

**Table 1:** List of primers used in this study.

Target genes	Primer sequences	Accession no.
NGAL	F: 5′- GACCTAGTAGCTGCTGAAACC -3′ R: 5′- GAGGATGGAAGTGACGTTGTAG -3′	NM_130741
KIM-1	F: 5′- TCCACACATGTACCAACATCAA -3′ R: 5′- GTCACAGTGCCATTCCAGTC -3′	NM_001161356
TNF-*α*	F: 5′-GACGTGGAACTGGCAGAAGAG-3′ R: 5′-CCGCCTGGAGTTCTGGAA-3′	NM_013693
IL-6	F: 5′-CCAGAGATACAAAGAAATGATGG-3′ R: 5′-ACTCCAGAAGACCAGAGGAAAT-3′	NM_031168
IL-1*β*	F: 5′- GCAACTGTTCCTGAACTCAACT -3′ R: 5′- ATCTTTTGGGGTCCGTCAACT -3′	NM_008361
GAPDH	F: 5′-ACTCCACTCACGGCAAATTC-3′ R: 5′-TCTCCATGGTGGTGAAGACA-3′	NM_001289726

## Data Availability

The data used to support the findings of this study are included within the article.
